# Synthesis of 5-Alkoxythieno[2,3-*e*][1,2,4]triazolo[4,3-*c*]pyrimidine Derivatives and Evaluation of Their Anticonvulsant Activities

**DOI:** 10.3390/molecules20046827

**Published:** 2015-04-15

**Authors:** Shi-Ben Wang, Guang-Chun Piao, Hong-Jian Zhang, Zhe-Shan Quan

**Affiliations:** Key Laboratory of Natural Resources and Functional Molecules of the Changbai Mountain, Affiliated Ministry of Education, College of Pharmacy, Yanbian University, Yanji 133000, China; E-Mails: 2010020146@ybu.edu.cn (S.-B.W.); 2012010623@ybu.edu.cn (G.-C.P.); 2012001040@ybu.edu.cn (H.-J.Z.)

**Keywords:** synthesis, triazole, maximal electroshock, neurotoxicity, pentylenetetrazole

## Abstract

This work concerns the design and synthesis of novel, substituted 5-alkoxythieno[2,3-*e*][1,2,4]triazolo[4,3-*c*]pyrimidine derivatives **5a–p** prepared from 3-amino-2-thiophenecarboxylic acid methyl ester. The final compounds were screened for their *in vivo* anticonvulsant activity using maximal electroshock (MES) and subcutaneous pentylenetetrazole (*sc*PTZ) tests. Neurotoxicity (NT) was tested using a rotarod test. The structure-anticonvulsant activity relationship analysis revealed that the most effective structural motif involves a substituted phenol, especially when substituted with a single chlorine, fluorine or trifluoromethyl group (at the *meta*-position), or two chlorine atoms. These molecules possessed high activity according to the MES and *sc*PTZ models. Quantitative assessment of the compounds after intraperitoneal administration in mice showed that the most active compound was 5-[3-(trifluoromethyl)phenoxy]thieno[2,3-*e*] [1,2,4]triazolo[4,3-*c*]pyrimidine (**5o**) with ED_50_ values of 11.5 mg/kg (MES) and 58.9 mg/kg (*sc*PTZ). Furthermore, compound **5o** was more effective in the MES and *sc*PTZ tests than the well-known anticonvulsant drugs carbamazepine and ethosuximide.

## 1. Introduction

Epilepsy is one of the most common disorders of the human brain, affecting more than 60 million individuals worldwide [1,2,3]. It has been observed that in as many as 25% of cases currently available antiepileptic drugs (AEDs) are unable to control the seizures [4]. Additionally, in many cases the clinical use of AEDs is restricted by their side effects such as gastrointestinal disturbances, gingival hyperplasia, headaches, nausea, anorexia, ataxia, hepatotoxicity, drowsiness, attention deficit, and cognitive problems [5,6,7,8,9]. Therefore, there is a ongoing need for the discovery of new chemical entities for the development of effective and safer AEDs. 

**Figure 1:**
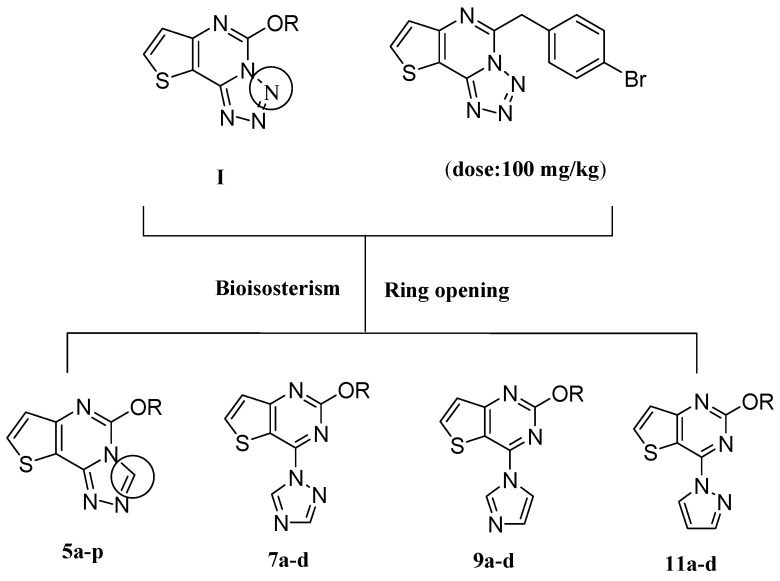
Structures of compounds **I** and target compounds.

Triazole derivatives have attracted continued interest over the years because of their diverse biological activities. These include anticonvulsant [10,11,12,13], antimicrobial [14,15] and enzyme inhibition effects [16,17]. Previous research in our laboratory led to the discovery of a series of derivatives of 5-alkoxytetrazolo[1,5-*c*]thieno[2,3-*e*]pyrimidine (**I**) having anticonvulsant activities [18]. The most effective among them was 5-(4-bromophenoxy)tetrazolo[1,5-*c*]thieno[2,3-*e*]pyrimidine, which showed anticonvulsant activity at a dose of 100 mg/kg. Based on the anticonvulsant activity of compound series **I**, we designed and synthesized a series of 5-alkoxythieno[2,3-*e*][1,2,4] triazolo[4,3-*c*] pyrimidines **5a**–**p** where one of the nitrogen atoms in the five-membered ring in the general structure of compound series **I** was replaced with a carbon (Figure 1). To our great satusfaction, the anticonvulsant activity of compounds **5a**–**p** was greatly improved. 

In order to discover whether a triazole ring that is not fused to the thieno[3,2-*d*]pyrimidine ring still has antiepileptic activity, another series of 2-alkoxy-4-(1*H*-1,2,4-triazol-1-yl)thieno[3,2-*d*]pyrimidine compounds **7a**–**d** was designed and synthesized through the ring-opening of compounds **5a**–**p**. For the purposes of investigating the structure-activity relationship, the triazole ring in compounds **7a**–**d** was replaced by other heterocycles such as imidazole, or pyrazole rings to give 2-alkoxy-4-(1*H*-imidazol-1-yl)thieno[3,2-*d*]pyrimidine **9a**–**d** or 2-alkoxy-4-(1*H*-pyrazol-1-yl)thieno[3,2-*d*]pyrimidine derivatives **11a**–**d**. Their structures were characterized using IR, ^1^H-NMR, HRMS, and ^13^C-NMR techniques.

The anticonvulsant activity of these compounds was evaluated using the maximal electroshock (MES) and subcutaneous pentylenetetrazole (*sc*PTZ) tests in mice, and their neurotoxicity (NT) was evaluated using the rotarod test. 

## 2. Results and Discussion

### 2.1. Chemistry

**Scheme 1:**
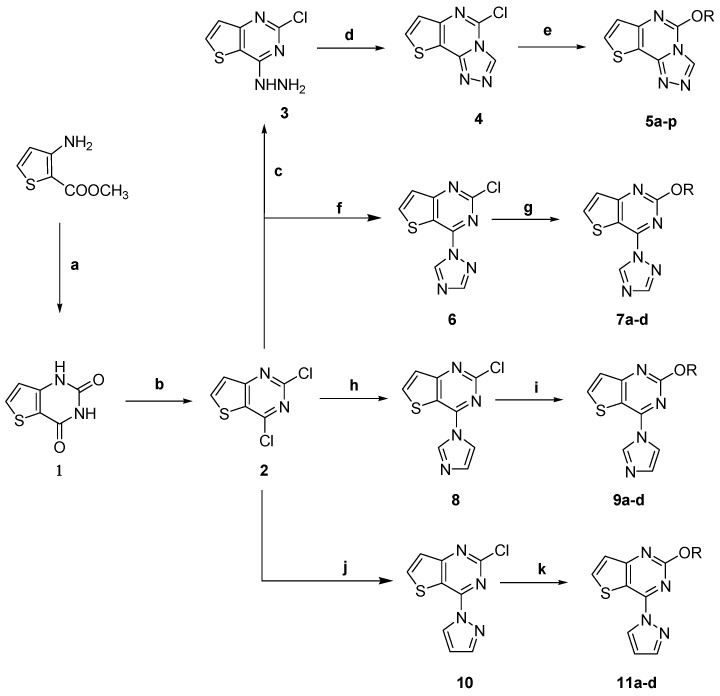
Synthetic routes to the target compounds.

The target compounds **5a**–**p** were synthesized as shown in Scheme 1. The starting materials 3-amino-2-thiophenecarboxylic acid methyl ester and urea were heated at 200 °C for 1.5 h to obtain compound **1**. This compound reacted further by refluxing with POCl_3_ to yield compound **2**. Compound **2** was reacted with hydrazine hydrate in methanol to afford the compound **3** in high yield [18]. Compound **4** was obtained via the cyclization of compound **3** with triethyl orthoformate [19]. Finally, the target compounds **5a**–**p** were obtained by reacting compound **4** with the appropriate substituted phenols in acetonitrile in the presence of K_2_CO_3_.

*Reagents and conditions*: (a) NH_2_CONH_2_, 180 °C, 1.5 h; (b) POCl_3_, 110 °C, 5 h; (c) NH_2_NH_2_.H_2_O, 50 °C, 1 h; (d) HC(OC_2_H_5_)_3_, reflux, 3 h; (e) ROH, CH_3_CN, reflux, 4 h; (f, h, j) 1,2,4-triazole/ imidazole/pyrazole, K_2_CO_3_, DMF, reflux, 3 h; (g, i, k) ROH, K_2_CO_3_, DMF, 80 °C, 10 h.

When 2,4-dichlorothieno[3,2-d]pyrimidine were refluxed in *N,N-*dimethylformamide (DMF) with different azoles such as triazole, imidazole and pyrazole in the presence of K_2_CO_3_ [20,21], the 4-chlorine atom of **1** was easily substituted by these heterocycles, producing the corresponding thienopyrimidines 2-chloro-4-(1*H*-1,2,4-triazol-1-yl)thieno[3,2-*d*]pyrimidine (**6**), 2-chloro-4-(1*H*-imidazol-1-yl)thieno[3,2-*d*]pyrimidine (**8**), and 2-chloro-4-(1*H*-pyrazol-1-yl)thieno[3,2-*d*]pyrimidine (**10**). The target compounds **7a**–**d**, **9a**–**d**, and **11a**–**d** were obtained by reacting compounds **6**, **8**, and **10** with a substituted phenol in DMF in the presence of K_2_CO_3_. The structures of the target compounds were characterized by spectroscopic methods. All spectral data corroborated the assumed structures.

### 2.2. Pharmacology

#### 2.2.1. Anticonvulsant Activity

The synthesized compounds were submitted to *in vivo* evaluation using the methods described in the Antiepileptic Drug Development Program (ADD) of the US National Institutes of Health according to previously described testing procedures [22,23]. The pharmacological evaluation was accepted by the Ethics Commission of China. Primary anticonvulsant studies involved two tests: maximal electroshock seizure (MES) and subcutaneous metrazol (*sc*MET), in mice. It is emphasized that nearly all clinically significant AEDs are effective in at least one of these two models, making them very useful tools for initial high throughput screening of candidate anticonvulsants. The MES test employs an electrical stimulus to induce generalized tonic clonic seizures and is used to identify compounds that prevent the spread of seizures. The scMET model utilizes chemically induced myoclonic seizures and recognizes the agents that are effective because they raise the seizure threshold. In addition to the primary anticonvulsant evaluation in the MES and scMET models, the neurotoxicity was assessed using a rotarod test. 

The compounds were administered intraperitoneally to mice at doses of 30, 100, and 300 mg/kg and tests were carried out 0.5 and 4 h after administration. The reference drugs, carbamazepine (for MES and rotarod test) and ethosuximide (for *sc*PTZ test) were used as positive controls. In preliminary screening, all of the newly synthesized compounds exhibited some degree of anti-MES activity. The protection offered by these compounds was indicative of their pharmacological ability to reduce seizure spread at a certain dose level. The results obtained after investigating the anticonvulsant activity of the synthesized compounds **5a**–**p**, **7a**–**d**, **9a**–**d**, and **11a**–**d** are summarized in Table 1 and Table 2.

For the **5a**–**p** series, all of the compounds were active in the MES test, which indicates their ability to prevent seizure spread. At a dose of 100 mg/kg, most of the compounds showed protection, except **5a**, **5j**, and **5m**–**n**. Five compounds, **5c**–**d**, **5f**, and **5o**–**p** showed protection against MES-induced seizures at a dose of 30 mg/kg. In these derivatives, only four compounds (**5i**, **5j**, **5l**, and **5n**) did not show activity 4 h after administration. Only two compounds showed significant neurotoxicity at a dose of 100 mg/kg, and six compounds were not neurotoxic at a dose of 300 mg/kg. Compounds **5d**, **5f**, **5h**, and **5o**–**p** were not neurotoxic at a dose of 300 mg/kg in 4 h. 

**Table 1:** Anticonvulsant activities of compounds **5a**–**p** in MES and *sc*PTZ tests. 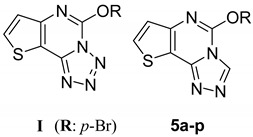

Compound	R	MES ^a^		*sc*PTZ ^b^		TOX ^c^	
		0.5 h	4 h	0.5 h	4 h	0.5 h	4 h
**5a**	-C_6_H_5_	300	300	-	-	300	-
**5b**	-C_6_H_4_(*o*-Cl)	100	100	300	-	-	-
**5c**	-C_6_H_4_(*p*-Cl)	30	100	100	300	300	-
**5d**	-C_6_H_4_(*m*-Cl)	30	100	100	-	100	300
**5e**	-C_6_H_4_(*o*-F)	100	300	-	-	-	-
**5f**	-C_6_H_4_(*m*-F)	30	100	100	300	300	300
**5g**	-C_6_H_4_(*p*-F)	100	100	100	300	-	-
**5h**	-C_6_H_4_(*o*-CH_3_)	100	100	-	-	300	300
**5i**	-C_6_H_4_(*m*-CH_3_)	100	-	-	-	-	-
**5j**	-C_6_H_4_(*p*-CH_3_)	300	-	-	-	300	-
**5k**	-C_6_H_4_(*o*-OCH_3_)	100	100	300	-	-	-
**5l**	-C_6_H_4_(*m*-OCH_3_)	100	-	-	-	300	-
**5m**	-C_6_H_4_(*p*-OCH_3_)	300	300	-	-	-	-
**5n**	-C_6_H_4_(*p*-Br)	300	-	-	-	300	-
**5o**	-C_6_H_4_(*m*-CF_3_)	30	100	100	300	300	300
**5p**	-C_6_H_3_(*2,4*-Cl)	30	100	100	-	100	300
**I**	-	100	300	-	-	-	-

^a^ Maximal electroshock: doses of 30, 100 and 300 mg/kg were administrated intraperitoneally in mice. The animals were examined 0.5 h and 4 h after administration; ^b^ Subcutaneous pentylenetetrazole test; ^c^ Neurotoxicity screening—rotorod test; (-): no activity or neurotoxicity at 300 mg·kg^−1^.

As shown in Table 2, compound **7c** showed anticonvulsant activity at a low dose of 30 mg/kg, and four of the compounds showed no neurotoxicity after 0.5 h. Five compounds, **7b**–**c**, **9c**, and **11c**–**d** showed protection against MES-induced seizures when tested 4 h after administration, and only compounds **7a**, **7c**, and **9c** were neurotoxic at a dose of 300 mg/kg after 4 h. 

The anticonvulsant activity of the compounds according to the *sc*MET model in mice was markedly lower than that according to the MES screen. Among the compounds, five (**5c**, **5f**–**g**, **5o**, and **7c**) were active after both 0.5 h and 4 h. Only seven compounds (**5c**–**d**, **5f**–**g**, and **5o**–**p**) showed considerable levels of seizure protection (after 0.5 h) at a dose of 100 mg/kg.

Based on the preliminary biological data, the most active compounds **5c**, **5f**, **5o**, **5p**, and **7c** were chosen for quantification of the pharmacological parameters (ED_50_ and TD_50_) after i.p. administration to mice. The results for the new compounds along with the data for the standard AEDs (tested in the same conditions), carbamazepine and ethosuximide are shown in Table 3. 

**Table 2:** Anticonvulsant activities of compounds **7a**–**d**, **9a**–**d** and **11a**–**d** in MES and *sc*PTZ tests. 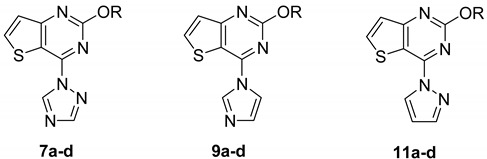

Compound	R	MES		*sc*PTZ		TOX	
		0.5 h	4 h	0.5 h	4 h	0.5 h	4 h
**7a**	-C_6_H_4_(*m*-Cl)	300	-	-	-	300	300
**7b**	-C_6_H_4_(*m*-F)	100	300	300	-	300	-
**7c**	-C_6_H_4_(*m*-CF_3_)	30	100	100	300	300	300
**7d**	-C_6_H_3_(*2,4*-Cl )	-	-	-	300	-	-
**9a**	-C_6_H_4_(*m*-Cl)	-	-	300	-	300	-
**9b**	-C_6_H_4_(*m*-F)	300	-	-	-	300	-
**9c**	-C_6_H_4_(*m*-CF_3_)	100	300	300	-	300	300
**9d**	-C_6_H_3_(*2,4*-Cl )	-	-	300	-	-	-
**11a**	-C_6_H_4_(*m*-Cl)	300	-	-	-	-	-
**11b**	-C_6_H_4_(*m*-F)	-	-	-	300	-	-
**11c**	-C_6_H_4_(*m*-CF_3_)	100	300	300	-	300	-
**11d**	-C_6_H_3_(*2,4*-Cl )	-	300	-	-	300	-

**Table 3:** Quantitative anticonvulsant data in mice (i.p.).

Compound	ED_50_ (MES) ^a^	ED_50_ (*sc*PTZ)	TD_50_ (NT) ^b^	PI ^c^	
				MES *sc*PTZ	
**5c**	18.5 (16.0–21.5)	85.2 (78.1–92.9)	294.6 (257.7–336.7)	15.8	3.4
**5f**	31.7 (23.6–42.5)	>100	328.6 (280.1–385.5)	10.3	<3.2
**5o**	11.5 (8.6–15.4)	58.9 (49.1–70.7)	204.6 (179.0–233.8)	17.7	3.4
**5p**	12.9 (12.9–23.2)	65.5 (52.5–72.9)	112.3 (89.8–140.4)	8.7	1.7
**7c**	30.4 (27.2–34.1)	71.6 (45.9–135)	237.1 (200.1–280.3)	7.8	3.3
**Carbamazepine**	11.8 (8.5–16.4)	–	76.1 (55.8–103.7)	6.4	-
**Ethosuximide**	–	106 (93–121)	343 (311–378)		3.2

^a^ ED_50_: median effective dose affording anticonvulsant protection in 50% of animals, the dose is measured in mg/kg; ^b^ TD_50_: median toxic dose eliciting minimal neurological toxicity in 50% of animals, the dose is measured in mg/kg; ^c^ PI: protective index (TD_50_/ED_50_); A dash indicates means—not tested.

The analysis of the MES quantitative data revealed lower activity of **5c**, **5f**, **5p**, and **7c** than carbamazepine, which is used as a reference antiepileptic drug in the MES model. At the same time, all of these molecules provided higher anti-MES protection in comparison with ethosuximide. The most effective compound according to the MES test was **5o** with an ED_50_ of 11.5 mg/kg. Despite lower activity in comparison with carbamazepine, **5o** was less neurotoxic and yielded a more favorable protective index (PI) of 17.7 than the reference drug’s PI of 6.4. Significantly, stronger activity was observed in the PTZ-induced seizures. In this test, the most effective were **5c**, **5o**, and **7c** with ED_50_ values and protective indexes much more favorable than ethosuximide (model AED for PTZ-induced seizures).

To determine the oral time of peak effect (TPE) of compounds **5c** and **5o**, we conducted a time-course test; compounds **5c** and **5o** reached the TPE at 1.5 h after oral administration. Next, we evaluated the anticonvulsant activity of compounds **5c** and **5o** against MES-induced seizures and neurotoxicity after oral administration to mice (Table 4), using carbamazepine as a reference. Compounds **5c** and **5o** showed significant oral activity against MES-induced seizures in mice, with ED_50_ values of 39.4 mg/kg and 28.4 mg/kg, respectively. The standard, carbamazepine, showed an ED_50_ value of 27.3 mg/kg and a TD_50_ value of 328.6 mg/kg, resulting in a PI of 12.0 under the same conditions. Thus, compounds **5c** and **5o** were judged safer than the anticonvulsant drug carbamazepine in MES and TOX models.

**Table 4:** Pharmacological evaluation of compound **5c** and **5o** and carbamazepine administered orally to mice.

Compound	R	Time (h)	ED_50_ (MES)	TD_50_ (NT)	PI
**5c**	-C_6_H_4_(*p*-Cl)	1.5	39.4 (34.0–45.6)	642.2 (609.7–689.6)	16.2
**5o**	-C_6_H_4_(*m*-CF_3_)	1.5	28.4 (23.1–32.6)	510.5 (484.4–648.5)	17.9
**CBZ**	-	1.5	27.3(19.7–37.9)	328.6 (229.9–469.7)	12.0

#### 2.2.2. Structure-Activity Relationship

In this study, we prepared four series of compounds and evaluated their preliminary anticonvulsant activities, and we established the following structure-activity relationships (SARs). Analysis of the anticonvulsant activities of compounds **5a**–**p** resulted in the establishment of several SARs. Among the phenoxy-substituted derivatives, the position of the substituent group on the benzene ring appeared to greatly influence the anticonvulsant activity, especially with a chlorine, fluorine, trifluoromethyl group (in the *meta*-position), or two chlorine atoms. These molecules showed high activity in the MES and/or the *sc*PTZ models. Changing the position of the chlorine atom to the *ortho* or *para* position as well as introduction of methyl and methoxy groups decreased the anticonvulsant activity. The same effect was observed for unsubstituted derivative **5a**, which protects against MES seizures only at dose of 300 mg/kg. When the triazole ring of compounds **5c**, **5f**, **5o**, and **5p** was opened, their anticonvulsant activities were weaker, and the PIs were lower for **7a**–**d** compared to these compounds. When the triazole ring in compounds **7a**–**d** was replaced with other heterocycles (*i.e.*, imidazole and pyrazole), the resultant compounds **9a**–**d** and **11a**–**d** had slightly decreased activity as compared to the compounds containing the triazole ring. These data indicate that separating the triazole from the thieno[3,2-*d*]pyrimidine, instead of incorporating the triazole into the thieno[3,2-*d*]pyrimidine, leads to weaker anticonvulsant activity and lower PI.

## 3. Experimental Section

### 3.1. General Information

Melting points were determined in open capillary tubes and were uncorrected. IR spectra were recorded (in KBr) on an IRPrestige-21 (Perkin Elmer, Waltham, MA, USA). ^1^H-NMR and ^13^C-NMR spectra were measured on an AV-300 spectrometer (Bruker, Flawil, Switzerland) with all chemical shifts given in ppm relative to tetramethylsilane. Mass spectra were measured on an HP1100LC (Agilent Technologies, Palo Alto, CA, USA) system. High-resolution mass spectra were measured on a MALDI-TOF/TOF mass spectrometer (Bruker Daltonik, Bremen, Germany). The principal chemicals were purchased from Aldrich Chemical (Shenyang, China).

### 3.2. Chemistry

#### 3.2.1. Synthesis of Thieno[3,2-*d*]pyrimidine-2,4(1*H*,3*H*)-dione (**1**)

3-Amino-2-thiophenecarboxylic acid methyl ester (1 g, 6.36 mmol) and urea (3 g, 50 mmol) were placed in a round bottomed flask, and the mixture was heated at 200 °C for 1.5 h. After the reaction was complete, the residue was dissolved in 20% NaOH (40 mL) and a solution of 10% HCl was added to adjust the pH to 5–6. The resulting solid was filtered, washed with water and dried. Yield: 79%, mp: 311–314 °C. ^1^H-NMR (CDCl_3_) δ: 6.89 (t, 1H, *J* = 3.00 Hz, S-C=C-H), 8.02 (t, 1H, *J* = 3.00 Hz, S-C-H), 11.19 (s, 1H, -CONH), 11.54 (s, 1H, -CONHCO-). MS-EI *m*/*z* 169 (M + 1).

#### 3.2.2. Synthesis of 2,4-Dichlorothieno[3,2-*d*]pyrimidine (**2**)

Compound **1** (1 g, 5.9 mmol) was dissolved in phosphorus oxychloride (15 mL) and stirred under reflux for 5 h. Two-thirds of the solvent was removed under vacuum. The mixture was poured into ice-water (50 mL). The precipitate that separated was collected by filtration, washed with water, and dried to give compound **2** as a white solid. Yield: 74%, mp: 140–143 °C. ^1^H-NMR (CDCl_3_) δ: 7.55 (d, 1H, *J* = 6.00 Hz, S-C=C-H), 8.13 (d, 1H, *J* = 6.00 Hz, S-C-H). MS-EI *m*/*z* 205 (M + 1).

#### 3.2.3. Synthesis of 1-(2-Chlorothieno[3,2-*d*]pyrimidin-4-yl)hydrazine (**3**)

To a solution of hydrazine hydrate (0.6 mL) in methanol (5 mL), a solution of 2,4-dichlorothieno [3,2-*d*]pyrimidine (4.9 mmol) in methanol (30 mL) was added and the reaction mixture was heated at 50 °C for 1 h. The solvent was concentrated under reduced pressure then diluted with an petroleum ether. The precipitated solid product was filtered, and did not require additional purification. Yield: 84%, mp: 314–316 °C. ^1^H-NMR (CDCl_3_) δ: 5.01 (s, 2H, -NH_2_), 7.23 (d, 1H, *J* = 6.00 Hz, S-C=C-H), 8.13 (d, 1H, *J* = 6.00 Hz, S-C-H), 9.48 (s, 1H, -NH). MS-EI *m*/*z* 201 (M + 1).

#### 3.2.4. Synthesis of 5-Chlorothieno[2,3-*e*][1,2,4]triazolo[4,3-*c*]pyrimidine (**4**)

A mixture of compound **3** (0.8 g, 4.0 mmol) and triethyl orthoformate (15 mL) was heated at 130 °C for 3 h. After removing the excess triethyl orthoformate under reduced pressure, water (10 mL) was added; the resulting precipitate was filtered and washed with water to produce **4** as a white solid. Yield: 84%, mp: 201–203 °C. ^1^H-NMR (CDCl_3_) δ: 7.56 (d, 1H, *J* = 6.00 Hz, S-C=C-H), 7.83 (d, 1H, *J* = 6.00 Hz, S-C-H), 8.99 (s, 1H, triazole-H). MS-EI *m*/*z* 211 (M + 1).

#### 3.2.5. General Procedure for the Synthesis of 5-Alkoxythieno[2,3-*e*][1,2,4]triazolo[4,3-*c*]pyrimidine Derivatives **5a**–**p**

K_2_CO_3_ (2.5 mmol) and appropriately substituted phenol (2.5 mmol) were dissolved in acetonitrile (15 mL) and the mixture was heated and stirred for 30 min at 75 °C. 5-Chlorothieno[2,3-*e*][1,2,4] triazolo[4,3-*c*]pyrimidine (0.5 g, 2.4 mmol) was added to the reaction mixture. Reaction completion was monitored by TLC, the solvent was then evaporated under reduced pressure. Water (30 mL) was added; the precipitate was collected and recrystallized from ethanol. The yields and melting point data of each compound are given below.

*5-Phenoxythieno[2,3-e][1,2,4]triazolo[4,3-c]pyrimidine* (**5a**). White solid in 62%, mp: 183–184 °C. ^1^H-NMR (CDCl_3_) δ: 7.31–7.33 (d, 1H, *J* = 5.25 Hz, S-C=C-H), 7.38–7.57 (m, 5H, Ar-H), 7.69–7.70 (d, 1H, *J* = 5.25 Hz, S-C-H), 9.08 (s, 1H, triazole-H). ^13^C-NMR (CDCl_3_) δ: 113.14, 121.38, 121.38, 124.46, 126.94, 129.99, 129.99, 130.07, 133.27, 145.00, 147.61, 148.35, 151.05. IR (KBr) cm^−1^: 1637 (C=N). MS-EI *m*/*z* 269 (M + 1). ESI-HRMS calcd for C_13_H_8_N_4_OS^+^ ([M + H]^+^): 269.0419; found: 269.0412. 

*5-(2-Chlorophenoxy)thieno[2,3-e][1,2,4]triazolo[4,3-c]pyrimidine* (**5b**). White solid in 58%, mp: 211–213 °C. ^1^H-NMR (CDCl_3_) δ: 7.29–7.30 (d, 1H, *J* = 5.25 Hz, S-C=C-H), 7.35–7.59 (m, 4H, Ar-H), 7.68–7.70 (d, 1H, *J* = 5.22 Hz, S-C-H), 9.11 (s, 1H, triazole-H). ^13^C-NMR (CDCl_3_) δ: 113.44, 123.70, 124.45, 126.87, 128.15, 128.28, 130.17, 130.89, 133.23, 144.09, 147.10, 147.64, 148.21. IR (KBr) cm^−1^: 1635 (C=N). MS-EI *m*/*z* 303 (M + 1). ESI-HRMS calcd for C_13_H_7_ClN_4_OS^+^ ([M + H]^+^): 303.0029; found: 303.0022.

*5-(3-Chlorophenoxy)thieno[2,3-e][1,2,4]triazolo[4,3-c]pyrimidine* (**5c**). White solid in 70%, mp: 175–177 °C. ^1^H-NMR (CDCl_3_) δ: 7.32–7.34 (d, 1H, *J* = 5.22 Hz, S-C=C-H), 7.33–7.50 (m, 4H, Ar-H), 7.70–7.72 (d, 1H, *J* = 5.28 Hz, S-C-H), 9.04 (s, 1H, triazole-H). ^13^C-NMR (CDCl_3_) δ: 113.39, 119.82, 122.16, 124.40, 127.29, 130.29, 130.72, 133.11, 135.21, 144.51, 147.54, 148.06, 151.31. IR (KBr) cm^−1^: 1637 (C=N). MS-EI *m/z* 303 (M + 1). ESI-HRMS calcd for C_13_H_7_ClN_4_OS^+^ ([M + H]^+^): 303.0029; found: 303.0024.

*5-(4-Chlorophenoxy)thieno[2,3-e][1,2,4]triazolo[4,3-c]pyrimidine* (**5d**). White solid in 64%, mp: 200–202 °C. ^1^H-NMR (CDCl_3_) δ: 7.29–7.31 (d, 1H, *J* = 5.25 Hz, S-C=C-H), 7.34–7.50 (dd, 4H, Ar-H), 7.68–7.70 (d, 1H, *J* = 5.28 Hz, S-C-H), 9.03 (s, 1H, triazole-H). ^13^C-NMR (CDCl_3_) δ: 113.28, 122.88, 122.88, 124.35, 130.06, 130.06, 130.24, 132.40, 133.14, 144.70, 147.52, 148.09, 149.44. IR (KBr) cm^−1^: 1639 (C=N). MS-EI *m*/*z* 303 (M + 1). ESI-HRMS calcd for C_13_H_7_ClN_4_OS^+^ ([M + H]^+^): 303.0029; found: 303.0021.

*5-(2-Fluorophenoxy)thieno[2,3-e][1,2,4]triazolo[4,3-c]pyrimidine* (**5e**). White solid in 67%, mp: 191–193 °C. ^1^H-NMR (CDCl_3_) δ: 7.29–7.31 (d, 1H, *J* = 5.22 Hz, S-C=C-H), 7.30–7.48 (m, 4H, Ar-H), 7.69–7.71 (d, 1H, *J* = 5.25 Hz, S-C-H), 9.08 (s, 1H, triazole-H). ^13^C-NMR (CDCl_3_) δ: 113.48, 117.21, 123.58, 124.41, 125.00, 128.28, 130.18, 133.20, 138.47, 144.13, 147.61, 148.17, 152.37. IR (KBr) cm^−1^: 1636 (C=N). MS-EI *m*/*z* 287 (M + 1). ESI-HRMS calcd for C_13_H_7_FN_4_OS^+^ ([M + H]^+^): 287.0325; found: 287.0319.

*5-(3-Fluorophenoxy)thieno[2,3-e][1,2,4]triazolo[4,3-c]pyrimidine* (**5f**). White solid in 58%, mp: 182–183 °C. ^1^H-NMR (CDCl_3_) δ: 7.33–7.35 (d, 1H, *J* = 5.25 Hz, S-C=C-H), 7.10–7.55 (m, 4H, Ar-H), 7.71–7.73 (d, 1H, *J* = 5.28 Hz, S-C-H), 9.05 (s, 1H, triazole-H). ^13^C-NMR (CDCl_3_) δ: 109.60, 113.46, 114.26, 124.40, 130.26, 130.86, 133.12, 144.47, 147.58, 148.06, 151.53, 161.35, 164.65. IR (KBr) cm^−1^: 1637 (C=N). MS-EI *m/z* 287 (M + 1). ESI-HRMS calcd for C_13_H_7_FN_4_OS^+^ ([M + H]^+^): 287.0325; found: 287.0322.

*5-(4-Fluorophenoxy)thieno[2,3-e][1,2,4]triazolo[4,3-c]pyrimidine* (**5g**). White solid in 62%, mp: 193–194 °C. ^1^H-NMR (CDCl_3_) δ: 7.29–7.31 (d, 1H, *J* = 5.25 Hz, S-C=C-H), 7.18–7.40 (dd, 4H, Ar-H), 7.68–7.70 (d, 1H, *J* = 5.28 Hz, S-C-H), 9.04 (s, 1H, triazole-H). ^13^C-NMR (CDCl_3_) δ: 113.21, 116.57, 116.88, 122.98, 124.37, 130.18, 133.17, 144.99, 146.76, 147.55, 148.18, 159.14, 162.40. IR (KBr) cm^−1^: 1635 (C=N). MS-EI *m/z* 287 (M + 1). ESI-HRMS calcd for C_13_H_7_FN_4_OS^+^ ([M + H]^+^): 287.0325; found: 287.0317.

*5-(2-Methylphenoxy)thieno[2,3-e][1,2,4]triazolo[4,3-c]pyrimidine* (**5h**). White solid in 57%, mp: 184–186 °C. ^1^H-NMR (CDCl_3_) δ: 2.26 (s, 1H, -CH_3_), 7.30–7.32 (d, 1H, *J* = 5.22 Hz, S-C=C-H), 7.29–7.35 (m, 4H, Ar-H), 7.66–7.68 (d, 1H, *J* = 5.25 Hz, S-C-H), 9.08 (s, 1H, triazole-H). ^13^C-NMR (CDCl_3_) δ: 16.19, 112.97, 121.72, 124.50, 127.13, 127.47, 130.04, 130.23, 131.75, 133.22, 144.60, 147.65, 148.53, 149.67. IR (KBr) cm^−1^: 1637 (C=N). MS-EI *m*/*z* 283 (M + 1). ESI-HRMS calcd for C_14_H_10_N_4_OS^+^ ([M + H]^+^): 283.0575; found: 283.0569.

*5-(3-Methylphenoxy)thieno[2,3-e][1,2,4]triazolo[4,3-c]pyrimidine* (**5i**). White solid in 72%, mp: 176–178 °C. ^1^H-NMR (CDCl_3_) δ: 2.45 (s, 1H, -CH_3_), 7.32–7.34 (d, 1H, *J* = 5.22 Hz, S-C=C-H), 7.19–7.43 (m, 4H, Ar-H), 7.68–7.70 (d, 1H, *J* = 5.22 Hz, S-C-H), 9.06 (s, 1H, triazole-H). ^13^C-NMR (CDCl_3_) δ: 21.46, 113.08, 118.29, 121.83, 124.50, 127.74, 129.65, 130.00, 133.28, 140.38, 145.09, 147.61, 148.41, 151.01. IR (KBr) cm^−1^: 1633 (C=N). MS-EI *m*/*z* 283 (M + 1). ESI-HRMS calcd for C_14_H_10_N_4_OS^+^ ([M + H]^+^): 283.0575; found: 283.0567.

*5-(4-Methylphenoxy)thieno[2,3-e][1,2,4]triazolo[4,3-c]pyrimidine* (**5j**). White solid in 62%, mp: 180–182 °C. ^1^H-NMR (CDCl_3_) δ: 2.45 (s, 1H, -CH_3_), 7.31–7.33 (d, 1H, *J* = 5.40 Hz, S-C=C-H), 7.24–7.34 (dd, 4H, Ar-H), 7.68–7.69 (d, 1H, *J* = 5.28 Hz, S-C-H), 9.06 (s, 1H, triazole-H). ^13^C-NMR (CDCl_3_) δ: 21.03, 113.06, 121.03, 121.03, 124.49, 129.98, 130.47, 130.47, 133.29, 136.76, 145.21, 147.62, 148.43, 148.86. IR (KBr) cm^−1^: 1633 (C=N). MS-EI *m*/*z* 283 (M + 1). ESI-HRMS calcd for C_14_H_10_N_4_OS^+^ ([M + H]^+^): 283.0575; found: 283.0570.

*5-(2-Methoxyphenoxy)thieno[2,3-e][1,2,4]triazolo[4,3-c]pyrimidine* (**5k**). White solid in 66%, mp: 168–170 °C. ^1^H-NMR (CDCl_3_) δ: 3.76 (s, 1H, -OCH_3_), 7.28–7.30 (d, 1H, *J* = 5.28 Hz, S-C=C-H), 7.07–7.37 (m, 4H, Ar-H), 7.66–7.68 (d, 1H, *J* = 5.28 Hz, S-C-H), 9.08 (s, 1H, triazole-H). ^13^C-NMR (CDCl_3_) δ: 55.88, 112.92, 113.96, 121.01, 122.70, 124.50, 127.95, 129.85, 133.49, 140.04, 144.91, 147.67, 148.68, 151.05. IR (KBr) cm^−1^: 1637 (C=N). MS-EI *m*/*z* 299 (M + 1). ESI-HRMS calcd for C_14_H_10_N_4_O_2_S^+^ ([M + H]^+^): 299.0524; found: 299.0518.

*5-(3-Methoxyphenoxy)thieno[2,3-e][1,2,4]triazolo[4,3-c]pyrimidine* (**5l**). White solid in 69%, mp: 177–178 °C. ^1^H-NMR (CDCl_3_) δ: 3.86 (s, 1H, -OCH_3_), 7.31–7.33 (d, 1H, *J* = 5.28 Hz, S-C=C-H), 6.91–7.44 (m, 4H, Ar-H), 7.68–7.70 (d, 1H, *J* = 5.25 Hz, S-C-H), 9.05 (s, 1H, triazole-H). ^13^C-NMR (CDCl_3_) δ: 55.61, 107.56, 112.54, 113.13, 113.40, 124.48, 130.06, 130.34, 133.25, 144.87, 147.58, 148.35, 151.89, 160.82. IR (KBr) cm^−1^: 1633 (C=N). MS-EI *m*/*z* 299 (M + 1). ESI-HRMS calcd for C_14_H_10_N_4_O_2_S^+^ ([M + H]^+^): 299.0524; found: 299.0514.

*5-(4-Methoxyphenoxy)thieno[2,3-e][1,2,4]triazolo[4,3-c]pyrimidine* (**5m**). White solid in 71%, mp: 180–182 °C. ^1^H-NMR (CDCl_3_) δ: 3.87 (s, 1H, -OCH_3_), 7.29–7.31 (d, 1H, *J* = 5.22 Hz, S-C=C-H), 7.00–7.31 (dd, 4H, Ar-H), 7.66–7.68 (d, 1H, *J* = 5.22 Hz, S-C-H), 9.04 (s, 1H, triazole-H). ^13^C-NMR (CDCl_3_) δ: 55.68, 112.97, 114.85, 114.85, 122.26, 122.26, 124.46, 129.99, 133.28, 144.46, 145.40, 147.58, 148.43, 157.97. IR (KBr) cm^−1^: 1635 (C=N). MS-EI *m*/*z* 299 (M + 1). ESI-HRMS calcd for C_14_H_10_N_4_O_2_S^+^ ([M + H]^+^): 299.0524; found: 299.0523.

*5-(4-Bromophenoxy)thieno[2,3-e][1,2,4]triazolo[4,3-c]pyrimidine* (**5n**). White solid in 40%, mp: 193–184 °C. ^1^H-NMR (CDCl_3_) δ: 7.43–7.45 (d, 1H, *J* = 5.28 Hz, S-C=C-H), 7.16–7.86 (dd, 4H, Ar-H), 7.77–7.79 (d, 1H, *J* = 5.28 Hz, S-C-H), 8.36 (s, 1H, triazole-H). ^13^C-NMR (CDCl_3_) δ: 111.60, 115.77, 116.08, 121.75, 121.85, 124.48, 131.13, 133.34, 141.27, 149.02, 151.54, 153.13, 157.74. IR (KBr) cm^−1^: 1639 (C=N). MS-EI *m*/*z* 347 (M + 1). ESI-HRMS calcd for C_13_H_7_BrN_4_OS^+^ ([M + H]^+^): 346.9524; found: 346.9517.

*5-[3-(Trifluoromethyl)phenoxy]thieno[2,3-e][1,2,4]triazolo[4,3-c]pyrimidine* (**5o**). White solid in 57%, mp: 185–186 °C. ^1^H-NMR (CDCl_3_) δ: 7.32–7.34 (d, 1H, *J* = 5.25 Hz, S-C=C-H), 7.64–7.74 (m, 4H, Ar-H), 7.72–7.74 (d, 1H, *J* = 5.25 Hz, S-C-H), 9.08 (s, 1H, triazole-H). ^13^C-NMR (CDCl_3_) δ: 113.50, 118.93, 123.77, 124.36, 125.12, 130.33, 130.66, 132.30, 132.74, 133.08, 144.45, 147.54, 147.94, 151.00. IR (KBr) cm^−1^: 1635 (C=N). MS-EI *m*/*z* 337 (M + 1). ESI-HRMS calcd for C_14_H_7_F_3_N_4_OS^+^ ([M + H]^+^): 337.0293; found: 337.0287.

*5-(2,4-Dichlorophenoxy)thieno[2,3-e][1,2,4]triazolo[4,3-c]pyrimidine* (**5p**). White solid in 60%, mp: 168–170 °C. ^1^H-NMR (CDCl_3_) δ: 7.30–7.32 (d, 1H, *J* = 5.22 Hz, S-C=C-H), 7.44–7.59 (m, 3H, Ar-H), 7.70–7.72 (d, 1H, *J* = 5.22 Hz, S-C-H), 9.09 (s, 1H, triazole-H). ^13^C-NMR (CDCl_3_) δ: 113.62, 124.36, 124.61, 127.87, 128.51, 130.37, 130.71, 133.12, 133.21, 143.82, 145.76, 147.60, 148.00. IR (KBr) cm^−1^: 1637 (C=N). MS-EI *m*/*z* 337 (M + 1). ESI-HRMS calcd for C_13_H_6_Cl_2_N_4_OS^+^ ([M + H]^+^): 336.9639; found: 336.9631.

#### 3.2.6. General Procedure for the Synthesis of 2-Alkoxy-4-(1*H*-1,2,4-triazol-1-yl)thieno[3,2-*d*]pyrimidine **7a**–**d**, 2-Alkoxy-4-(1*H*-imidazol-1-yl)thieno[3,2-*d*]pyrimidine **9a**–**d** and 2-Alkoxy-4-(1*H*-pyrazol-1-yl)thieno[3,2-*d*]pyrimidine Derivatives **11a**–**d**

A solution of **2** (2 mmol), 1,2,4-triazole/imidazole/pyrazole (2.2 mmol), and K_2_CO_3_ (2.2 mmol) in *N*,*N-*dimethylformamide (30 mL) was refluxed for 3 h. The reaction mixture was cooled to room temperature, concentrated by evaporation under vacuum, and white solids obtained were filtered, washed with water and dried. All the compounds were recrystallized in ethanol to produce the pure compounds **6**, **8**, and **10**. A mixture of an appropriately substituted phenol (5.1 mmol) and K_2_CO_3_ (5.1 mmol) was placed in a round-bottomed flask with *N*,*N-*dimethylformamide (15 mL). The mixture was stirred and heated at 80 °C for 0.5 h, and compound **6**, **8**, or **10** (1 g, 4.7 mmol) was added to the mixture. After the reaction was completed, the solvent was poured into ice water. The precipitate was filtered and washed with water to produce a crude product, and purified using silica-gel column chromatography with methanol-dichloromethane (*v*/*v* 1:60) to give a white solid. The yields and melting point data of each compound are given below. 

*2-(3-Chlorophenoxy)-4-(1H-1,2,4-triazol-1-yl)thieno[3,2-d]pyrimidine* (**7a**). White solid in 55%, mp: 156–158 °C. ^1^H-NMR (CDCl_3_) δ: 7.19–7.41 (m, 4H, Ar-H), 7.44–7.46 (d, 1H, *J* = 6.00 Hz, S-C=C-H), 8.16–8.18 (d, 1H, *J* = 6.00 Hz, S-C-H), 8.27 (s, 1H, triazole-H), 9.27 (s, 1H, triazole-H). ^13^C-NMR (CDCl_3_) δ: 113.79, 119.97, 122.27, 123.78, 125.86, 130.37, 134.84, 142.08, 142.86, 143.23, 151.10, 153.52, 153.71, 166.74. IR (KBr) cm^−1^: 1586 (C=N). MS-EI *m*/*z* 330 (M + 1). ESI-HRMS calcd for C_14_H_8_ClN_5_OS^+^ ([M + H]^+^): 330.0211; found: 330.0202.

*2-(3-Fluorophenoxy)-4-(1H-1,2,4-triazol-1-yl)thieno[3,2-d]pyrimidine* (**7b**). White solid in 61%, mp: 179–181 °C. ^1^H-NMR (CDCl_3_) δ: 7.02–7.48 (m, 4H, Ar-H), 7.44–7.46 (d, 1H, *J* = 6.00 Hz, S-C=C-H), 8.17–8.18 (d, 1H, *J* = 3.00 Hz, S-C-H), 8.28 (s, 1H, triazole-H), 9.28 (s, 1H, triazole-H). ^13^C-NMR (CDCl_3_) δ: 109.49, 112.46, 113.79, 117.31, 123.77, 130.30, 142.04, 142.85, 151.12, 153.71, 161.50, 161.99, 164.77, 166.75. IR (KBr) cm^−1^: 1589 (C=N). MS-EI *m*/*z* 314 (M + 1). ESI-HRMS calcd for C_14_H_8_FN_5_OS^+^ ([M + H]^+^): 314.0506; found: 314.0497.

*2-[3-(Trifluoromethyl)phenoxy]-4-(1H-1,2,4-triazol-1-yl)thieno[3,2-d]pyrimidine* (**7c**). White solid in 65%, mp: 127–129 °C. ^1^H-NMR (CDCl_3_) δ: 7.42–7.44 (d, 1H, *J* = 6.00 Hz, S-C=C-H), 7.51–7.59 (m, 4H, Ar-H), 8.16–8.18 (d, 1H, *J* = 6.00 Hz, S-C-H), 8.27 (s, 1H, triazole-H), 9.24 (s, 1H, triazole-H). ^13^C-NMR (CDCl_3_) δ: 113.90, 118.93, 122.28, 123.72, 125.13, 130.20, 131.90, 132.34, 142.19, 142.80, 151.10, 153.09, 161.87, 166.69. IR (KBr) cm^−1^: 1587 (C=N). MS-EI *m*/*z* 364 (M + 1). ESI-HRMS calcd for C_15_H_8_F_3_N_5_OS^+^ ([M + H]^+^): 364.0474; found: 364.0466.

*2-(2,4-Dichlorophenoxy)-4-(1H-1,2,4-triazol-1-yl)thieno[3,2-d]pyrimidine* (**7d**). White solid in 68%, mp: 206–208 °C. ^1^H-NMR (CDCl_3_) δ: 7.31–7.56 (m, 3H, Ar-H), 7.42–7.43(d, 1H, *J* = 3.00 Hz, S-C=C-H), 8.16–8.18 (d, 1H, *J* = 6.00 Hz, S-C-H), 8.27 (s, 1H, triazole-H), 9.26 (s, 1H, triazole-H). ^13^C-NMR (CDCl_3_) δ: 113.91, 123.74, 124.72, 128.21, 128.38, 130.38, 131.68, 142.13, 142.90, 147.81, 151.17, 153.72, 161.51, 166.73. IR (KBr) cm^−1^: 1589 (C=N). MS-EI *m*/*z* 364 (M + 1). ESI-HRMS calcd for C_14_H_7_Cl_2_N_5_OS^+^ ([M + H]^+^): 363.9821; found: 363.9814.

*2-(3-Chlorophenoxy)-4-(1H-imidazol-1-yl)thieno[3,2-d]pyrimidine* (**9a**). White solid in 62%, mp: 150–152 °C. ^1^H-NMR (CDCl_3_) δ: 7.17–7.31 (m, 4H, Ar-H), 7.37–7.42 (m, 1H, S-C=C-H), 7.42–7.51 (m, 1H, imidazole-H), 7.88–7.89 (d, 1H, imidazole-H), 8.08–8.10 (d, 1H, *J* = 6.00 Hz, S-C-H), 8.58 (s, 1H, imidazole-H). ^13^C-NMR (CDCl_3_) δ: 113.71, 116.91, 120.01, 122.29, 124.84, 125.82, 130.34, 131.68, 134.80, 136.27, 137.76, 151.70, 153.49, 162.64, 166.67. IR (KBr) cm^−1^: 1575 (C=N). MS-EI *m*/*z* 329 (M + 1). ESI-HRMS calcd for C_15_H_9_ClN_4_OS^+^ ([M + H]^+^): 329.0258; found: 329.0252.

*2-(3-Fluorophenoxy)-4-(1H-imidazol-1-yl)thieno[3,2-d]pyrimidine* (**9b**). White solid in 49%, mp: 168–170 °C. ^1^H-NMR (CDCl_3_) δ: 7.00–7.10 (m, 4H, Ar-H), 7.41–7.44 (d, 1H, *J* = 9.00 Hz, imidazole-H), 7.48–7.50 (d, 1H, *J* = 6.00 Hz, S-C=C-H), 7.90 (s, 1H, imidazole-H), 8.09–8.11 (d, 1H, *J* = 6.00 Hz, S-C-H), 8.59 (s, 1H, imidazole-H). ^13^C-NMR (CDCl_3_) δ: 109.51, 112.44, 113.71, 116.91, 124.83, 130.27, 131.68, 136.28, 137.73, 151.72, 153.81, 161.47, 162.62, 164.75, 166.68. IR (KBr) cm^−1^: 1577 (C=N). MS-EI *m*/*z* 313 (M + 1). ESI-HRMS calcd for C_15_H_9_FN_4_OS^+^ ([M + H]^+^): 313.0554; found: 313.0548.

*2-[3-(Trifluoromethyl)phenoxy]-4-(1H-imidazol-1-yl)thieno[3,2-d]pyrimidine* (**9c**). White solid in 57%, mp: 146–148 °C. ^1^H-NMR (CDCl_3_) δ: 7.47–7.49 (m, 2H, Ar-H), 7.58–7.59 (m, 2H, Ar-H; 1H, S-C=C-H; 1H, imidazole-H), 7.88 (s, 1H, imidazole-H), 8.09–8.11 (d, 1H, *J* = 6.00 Hz, S-C-H), 8.58 (s, 1H, imidazole-H). ^13^C-NMR (CDCl_3_) δ: 113.84, 116.89, 118.94, 119.00, 122.30, 124.80, 125.17, 130.18, 131.68, 132.31, 136.23, 137.87, 151.72, 153.08, 162.51, 166.66. IR (KBr) cm^−1^: 1575 (C=N). MS-EI *m*/*z* 363 (M + 1). ESI-HRMS calcd for C_16_H_9_F_3_N_4_OS^+^ ([M + H]^+^): 363.0522; found: 363.0513.

*2-(2,4-Dichlorophenoxy)-4-(1H-imidazol-1-yl)thieno[3,2-d]pyrimidine* (**9d**). White solid in 66%, mp: 144–146 °C. ^1^H-NMR (CDCl_3_) δ: 7.25–7.36 (m, 3H, Ar-H), 7.46–7.48(d, 1H, *J* = 6.00 Hz, S-C=C-H), 7.52–7.53 (d, 1H, imidazole-H), 7.86–7.87 (d, 1H, imidazole-H), 8.08–8.10 (d, 1H, *J* = 6.00 Hz, S-C-H), 8.55 (s, 1H, imidazole-H). ^13^C-NMR (CDCl_3_) δ: 113.85, 116.91, 116.91. 124.71, 124.79, 128.19, 128.37, 130.33, 131.68, 136.29, 137.87, 147.80, 151.73, 162.10, 166.69. IR (KBr) cm^−1^: 1576 (C=N). MS-EI *m*/*z* 363 (M + 1). ESI-HRMS calcd for C_15_H_8_Cl_2_N_4_OS^+^ ([M + H]^+^): 362.9869; found: 362.9861.

*2-(3-Chlorophenoxy)-4-(1H-pyrazol-1-yl)thieno[3,2-d]pyrimidine* (**11a**). White solid in 58%, mp: 183–185 °C. ^1^H-NMR (CDCl_3_) δ: 6.56–6.57 (m, 1H, pyrazole-H), 7.20–7.42 (m, 4H, Ar-H), 7.35–7.37 (m, 1H, S-C=C-H), 7.93 (s, 1H, pyrazole-H), 8.08–8.10 (d, 1H, *J* = 6.00 Hz, S-C-H), 8.59–8.60 (m, 1H, pyrazole-H). ^13^C-NMR (CDCl_3_) δ: 109.47, 113.57, 120.01, 122.29, 123.55, 125.48, 128.12, 130.22, 134.68, 141.48, 144.01, 153.21, 153.84, 162.10, 165.74. IR (KBr) cm^−1^: 1577 (C=N). MS-EI *m*/*z* 329 (M + 1). ESI-HRMS calcd for C_15_H_9_ClN_4_OS^+^ ([M + H]^+^): 329.0258; found: 329.0249.

*2-(3-Fluorophenoxy)-4-(1H-pyrazol-1-yl)thieno[3,2-d]pyrimidine* (**11b**). White solid in 53%, mp: 182–184 °C. ^1^H-NMR (CDCl_3_) δ: 6.56–6.57 (m, 1H, pyrazole-H), 6.98–7.46 (m, 4H, Ar-H), 7.39–7.40 (d, 1H, *J* = 3.00 Hz, S-C=C-H), 7.93 (s, 1H, pyrazole-H), 8.08–8.10 (d, 1H, *J* = 6.00 Hz, S-C-H), 8.59–8.60 (m, 1H, pyrazole-H). ^13^C-NMR (CDCl_3_) δ: 109.45, 109.80, 112.07, 113.57, 117.34, 123.55, 128.12, 130.13, 141.46, 144.01, 154.31, 161.47, 162.08, 164.74, 165.75. IR (KBr) cm^−1^: 1579 (C=N). MS-EI *m*/*z* 313 (M + 1). ESI-HRMS calcd for C_15_H_9_FN_4_OS^+^ ([M + H]^+^): 313.0554; found: 313.0545.

*2-[3-(Trifluoromethyl)phenoxy]-4-(1H-pyrazol-1-yl)thieno[3,2-d]pyrimidine* (**11c**). White solid in 67%, mp: 151–153 °C. ^1^H-NMR (CDCl_3_) δ: 6.55–6.56 (m, 1H, pyrazole-H), 7.37–7.39 (d, 1H, *J* = 6.00 Hz, S-C=C-H), 7.50–7.62 (m, 4H, Ar-H), 7.92 (m, 1H, pyrazole-H), 8.08–8.10 (d, 1H, *J* = 6.00 Hz, S-C-H), 8.55–8.56 (m, 1H, pyrazole-H). ^13^C-NMR (CDCl_3_) δ: 109.51, 113.67, 118.97, 129.91, 123.50, 125.15, 128.04, 130.04, 131.73, 132.16, 141.60, 144.05, 153.19, 153.40, 161.96, 165.72. IR (KBr) cm^−1^: 1579 (C=N). MS-EI *m*/*z* 363 (M + 1). ESI-HRMS calcd for C_16_H_9_F_3_N_4_OS^+^ ([M + H]^+^): 363.0522; found: 363.0515.

*2-(2,4-Dichlorophenoxy)-4-(1H-pyrazol-1-yl)thieno[3,2-d]pyrimidine* (**11d**). White solid in 45%, mp: 166–168 °C. ^1^H-NMR (CDCl_3_) δ: 6.55–6.56 (m, 1H, pyrazole-H), 7.31–7.38 (m, 3H, Ar-H), 7.53–7.54 (d, 1H, *J* = 3.00 Hz, S-C=C-H), 7.91–7.92 (m, 1H, pyrazole-H), 8.07–8.09 (d, 1H, *J* = 6.00 Hz, S-C-H), 8.56–8.57 (m, 1H, pyrazole-H). ^13^C-NMR (CDCl_3_) δ: 109.46, 113.65, 123.51, 124.84, 128.06, 128.17, 128.46, 130.26, 131.29, 141.54, 144.03, 148.09, 153.25, 161.66, 165.74. IR (KBr) cm^−1^: 1581 (C=N). MS-EI *m*/*z* 363 (M + 1). ESI-HRMS calcd for C_15_H_8_Cl_2_N_4_OS^+^ ([M + H]^+^): 362.9869; found: 362.9863.

### 3.3. Pharmacology

#### 3.3.1. Anticonvulsant Effects in the MES Test 

Seizures were induced in mice with a 60 Hz alternating current of 50 mA intensity. The current was applied via corneal electrodes for 0.2 s. Effective protection against the spread of MES-induced seizures was defined as the prevention of the hind leg and tonic maximal extension component of the seizure. 0.5 h and 4 h after the administration of the compounds, the activities were evaluated using an MES test [22,23].

#### 3.3.2. Neurotoxicity (NT) Screening 

The neurotoxicity of the compounds was measured in mice using the rotarod test. The mice were trained to stay on an accelerating rotarod of diameter 3.2 cm that rotates at 10 rpm. The trained animals were given intraperitoneal (i.p) injection of the test compounds. Neurotoxicity was indicated by the inability of the animals to maintain equilibrium on the rod for at least 1 min in each of the trials [22,23].

#### 3.3.3. The Subcutaneous Metrazol Seizure Test (scMET)

This screen utilizes a dose of pentylenetetrazole (85 mg/kg) that produces clonic seizures lasting for a period of at least five seconds in 97% (CD_97_) of animals tested. At the anticipated time of testing, the convulsant is administered subcutaneously. The test compound was administered intraperitoneally in mice, and the animals were observed over a 30 min period. Mice were tested at least two different time points (15 min, 30 min, 1 h, or 4 h) following i.p administration of 100 and 300 mg/kg of test compound. Absence of clonic spasms indicated a compound’s ability to counteract the effect of pentylenetetrazole on seizure threshold [24].

#### 3.3.4. Pharmacological Evaluation of Compounds **5c** and **5o** Administered Orally to Mice

The time-course effect of compounds **5c** and **5o** in the MES test was determined. A suspension of compounds **5c** and **5o** (50 mg/kg) in 0.5% methylcellulose was injected into mice by oral administration (p.o.). The mice were divided into six groups (*n* = 10). Subsequently, the animals were subjected to the MES test at various times: 0.5, 1, 1.5, 2, 2.5, and 3 h. The time of peak effect (TPE) was 1.5 h after the p.o. injection. Then, compounds **5c** and **5o** were evaluated for anticonvulsant activity against MES-induced seizures and oral neurotoxicity at its TPE when administered orally. This test involved the same procedures for determining ED_50_ and TD_50_ as used in the MES test and the TOX test screening, except that the test drug was administered orally to mice.

## 4. Conclusions

Based on our previous work, we designed and synthesized a series of 5-alkoxythieno[2,3-*e*] [1,2,4]triazolo[4,3-*c*]pyrimidine derivatives, we also evaluated their anticonvulsant activities using MES and PTZ tests. The results revealed that majority of compounds showed protective effects against seizures in the maximal electroshock tests (MES) and/or PTZ-induced seizures. Among the compounds synthesized, five compounds (**5c**, **5f**, **5o**, **5p**, and **7c**) were found to have promising anticonvulsant activity. In the MES screen, compound **5o** had an ED_50_ of 11.5 mg/kg and a TD_50_ of 204.6 mg/kg, resulting in a high PI value of 17.7 when compared to carbamazepine (PI = 6.4). The structure-anticonvulsant activity relationships analysis revealed that the most favorable structures are the substituted phenol, especially with single chlorine, fluorine, trifluoromethyl substituents (at the *meta*-position), or two chlorine atoms. These molecules possessed high activity according to the MES and/or *sc*PTZ models.
